# Predictors of length of hospital stay for preterm infants in Ethiopia: a competing risk analysis

**DOI:** 10.3389/fped.2023.1268087

**Published:** 2023-11-08

**Authors:** Zelalem Tazu Bonger, Biniyam Tedla Mamo, Sosna Bayu Birra, Alemayehu Worku Yalew

**Affiliations:** ^1^ Ohio State Global One Health Initiative, LLC, Addis Ababa, Ethiopia; ^2^ Department of Statistics, College of Natural and Computational Sciences, Addis Ababa University, Addis Ababa, Ethiopia; ^3^ School of Public Health, College of Health Sciences, Addis Ababa University, Addis Ababa, Ethiopia

**Keywords:** length of stay, cumulative incidence, competing risk, preterm infants, gestational age, low birth weight, newborn

## Abstract

**Background:**

Length of hospital stay (LOS) is one of the essential indicators for evaluating the efficiency and the quality-of-care service delivered. predicting LOS is critical for resource allocation, decision-making, lowering neonatal morbidity and death, enhancing clinical outcomes and parent counseling. In addition, extended hospital stays (long LOS_NICU) place a burden on the healthcare systems decreasing bed turnover rates as well as their financial stand and the mental stress on families. In Ethiopia, there is limited evidence on the determinant factors that influence on LOS.

**Objectives:**

To determine factors affecting neonatal intensive care unit length of stay for all preterm newborns who were discharged alive.

**Method:**

The study used a secondary data source, was collected for the Study of Illness in Preterm (SIP) infants project. The research study was a multicenter, cross-sectional, observational clinical study that took place in five Ethiopia hospitals from July 1, 2016, to May 31, 2018. The predictors of LOS were determined using Fine-Gray's competing risk analysis.

**Results:**

For this study 3,511 preterm infants admitted to the NICU were analyzed. About 28.8% of the preterm infants died during their time in neonatal care while 66.6% were discharged alive. At the end of the study 4.6% babies were still in the NICU. The overall median LOS (death or discharge) was 7 days, with an interquartile range of 8 days. The cumulative incidence of discharge rose with increasing in gestational age and birth weight, on the contrary, the rate of discharge was decreased by 45.7% with the development of RDS (SDH ratio: 0.543), by 75.9% with the development of apnea (SDH ratio: 0.241), by 36.2% with sepsis, and by 43.6% with pneumonia (SDH ratio: 0.564).

**Conclusions:**

Preterm newborns with a low gestational age and birth weight have a greater probability of having a prolonged LOS. Complications of the medical conditions RDS, apnea, sepsis, pneumonia, anemia, asphyxia, and NEC substantially raise LOS considerably.

## Introduction

Preterm birth, defined as giving birth before 37 weeks of gestation, is one of the leading causes of infant disease and mortality. It has been identified as a global concern, accounting for an estimated 11% of all live births globally (1). Annually, roughly 15 million infants are born prematurely and the number is rising globally (2). According to WHO, preterm can be further subdivided based on gestational age: extremely preterm (<28 weeks), very preterm (28–<32 weeks), and moderate to late preterm (32–<37 completed weeks of gestation) (3).

Access to quality care after delivery for admitted infants will have a greater role in improving their lives. Preterm newborns require special neonatal care due to their high risk of longer stays in the NICU. consequently, Predicting LOS in the NICU has gained attention in recent years. Length of hospital stay (LOS) is an essential index for assessing the efficiency of patient quality of care after delivery. Lowering the duration of hospital stay reduces the risk of nosocomial infection and medication adverse effects while additionally enhancing treatment quality. With Increasing LOS, the neonate can take a long duration of IV medication, a broad spectrum of antibiotics and other interventions that can increase the probability of having severe drug side effects and antibiotic resistance (4).

Predictions of the duration of stay are also required to support discussions between parents and physicians regarding the expected length of stay for a newborn (5). The capacity to reliably predict LOS in newborn care is critical for resource allocation, service commissioning, and assisting clinicians in their parent counseling (6). LOS among patients with the same condition or receiving the same type of surgical operation may differ due to complicated individual circumstances, differing procedural flows within various organizations, or divergences in medical practice. Prolonged LOS consumes more resources, hence focusing on controllable factors that impact LOS can enhance health system performance and minimize healthcare costs (7). Previous studies have frequently focused on the duration of stay for newborns who are released alive from neonatal care. The omission of newborns who die in neonatal care has been noted as a restriction of neonatal care length-of-stay studies (5, 8). Information on factors that affect LOS for preterm infants who were discharged alive by taking preterm babies who died in neonatal care into account is limited. The objective of the research was to identify the predictors of LOS in the NICU for all the preterm babies who were discharged alive.

## Material and methods

### Data

A secondary data source which was collected for the Study of Illness in Preterm (SIP) infants project. The SIP study was a prospective, multicenter, cross-sectional, observational clinical study conducted in five hospitals in Ethiopia from July 1, 2016, to May 31, 2018. Gondar University Hospital, Jimma University Hospital, and three Addis Abeba institutions (Gandhi Memorial Hospital, St Paul's Hospital Millennium Medical College and Tikur Ambessa Specilalized hospital included in this study. Methodological and other details of the SIP study have been published elsewhere (9, 10).

### Participants and study population

The study population was restricted to babies with an estimated gestational age of younger than 37 weeks with no congenital malformations or anomalies. Gestational age was defined here according to ultrasound measurements early in the second trimester. Data on the ultrasound examination was collected if available during the admission of the preterm infant from the hospital records. If no ultrasound data were available, gestational age was defined based on either the last menstrual period (LMP) or physical examination using the New Ballard Score (NBS).

If the difference between GA assessed by NBS and that calculated from LMP was not greater than 2 weeks, the last menstrual period GA was assumed to be correct and would be considered for this study. On the other hand, GA assessed by Ballard would be taken for this study if the difference between the two measurements was greater than 2 weeks (9). Preterm deliveries with missing data on any of the variables of interest were excluded from this study.

Anemia is defined here as hematocrit (HCT) that is more than 2 standard deviations below the age or less than the normal range for postnatal age and birth weight. For clinical purposes HCT < 45% in the 1st week of life (10).

Neonatal sepsis is defined as a clinical syndrome of bacteremia with systemic signs and symptoms of infection in the first 4 weeks of life (10).

RDS defined accordingly, respiratory distress syndrome occurs primarily in premature infants; its incidence is inversely related to gestational age and birth weight. Surfactant deficiency (decreased production and secretion) is the primary cause of RDS. clinical presented with, tachypnea, prominent (often audible) grunting, intercostal and subcostal retractions, nasal flaring, and cyanosis (11).

Jaundice (Hyperbilirubinemia) is defined as a newborn presents with yellowish discoloration of sclera, skin, mucus membranes and In newborns, jaundice appears when total bilirubin (TB) is more than 7 mg /dl. Neonatal jaundice can be classified as either physiologic or pathologic (10).

The diagnosis of hypoglycemia depends on the clinical setting but not solely on specific blood glucose level. For intervention or further evaluation, hypoglycemia could be defined as a blood glucose level less than 40 mg/dl (10).

Perinatal asphyxia is an insult to the fetus or newborn due to lack of oxygen (hypoxia) and/or a lack of perfusion (ischemia) to various organs (10).

### Outcome

The outcome variable of interest was the LOS of preterm infants who were discharged from the NICU. However, preterm infants who died in the NICU were considered as a competing event. Preterm infants who were still in the unit at the end of the follow-up were considered censored events. The number of days from the time of admission to the time of discharge was used to calculate the LOS.

### Predictors

Both demographic and clinical characteristics of the preterm newborn were considered as candidate predictors for the LOS of preterm infants. The demographic characteristics tested in the analyses were gestational age (<28, 28–31, 32–34, 35–36), sex (“Female/Male”) and birthplace (Home, transferred, study hospitals). Clinical characteristics included birth weight (less than 1,000 g, 1,000 g to less than 1,500 g, 1,500 g to less than 2,000 g, 2,000 g and above), maternal mode of delivery (“CS/Normal”), gestation type (“Multiple/Single”), respiratory distress syndrome (RDS), apnea, early onset neonatal sepsis, pneumonia, anemia, hyperbilirubinemia, feeding problems, hypoglycemia, asphyxia, necrotizing enterocolitis (NEC), and hypothermia. Maternal characteristics such as maternal age (19, 19–35, >35), pre-eclampsia, antenatal hemorrhage, maternal fever, and antenatal care received were also evaluated for candidacy.

### Explanatory data analysis

At the beginning of the analysis, the data was analysed in various ways (both tabular and graphical) to obtain details that could help make decisions about the next steps of the analysis. Histograms were used to explore the characteristics of the time until discharged alive and the time to death. The plot of cumulative incidence of follow-up time was used to illustrate the probabilities of in-hospital death and being discharged alive over time.

### Statistical analysis

To determine the predictors of LOS, a competing risk analysis proposed by Fine-Gray was used (12). The proposed regression modelling takes the presence of competing risk events into account through a multivariable regression analysis using semi-parametric proportional hazards. It has been developed by modelling the cumulative incidence function (CIF), which estimates the marginal probability for each competing event. The CIF is the best tool for predicting individual risks for the primary event. If we use 1 to denote the event of interest and 2 to denote the competing risk event, then the CIF for an event of interest can be formulated as:

$$CIF(t)\int_{0}^{t}S(s)h1(s)ds$$

where $S(t)=e^{-H_{1}(s)-H_{2}(s)}$ is the survival function at time s and is determined by both event of interest and the competing event.

The analysis is performed using the “crr” function under the “cmprsk” package in the R statistical software. As variable selection criteria for the Fine and Gray model, the stepwise regression procedure (forward and backward) was implemented using the R package “crrstep”.

## Results

**Figure 1:**
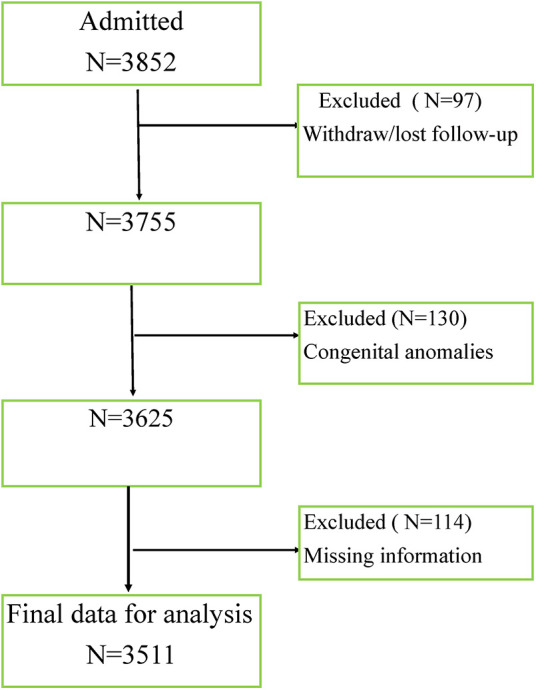
Screening of preterm infants admitted to NICU.

In the study period, a total of 3,852 preterm infants were admitted to the neonatal intensive care units of the study hospitals. Among these admitted preterm infants, 341 preterm infants were ineligible by the exclusion criteria as shown in Figure 1 and 3,511 newborns were studied in this research. Preterm deliveries with congenital deformity's and chromosomal anomalies were excluded from the study. Missing information on infant gender, birth weight and other variables of interest were also excluded from the study.

Summary characteristics of the preterm babies were analyzed associated with the outcome of interest is provided in Table 1. Among preterm neonates included in the study, 1,882 (53.6%) were male infants and 1,214 (34.6%) were multiple gestations. More than 37% of the babies admitted were born through cesarean section. About 28.8% of the preterm infants died during their time in neonatal care while 66.6% were discharged alive.

**Table 1:** Summary statistics of the preterm babies admitted in NICU from 2016 to 2018.

Variables	Categories	# of Preterm infants	Still in NICU (%)	Discharged alive (%)	Died prior to discharge (%)
Gestational age (in weeks)	<28	101	6.9	5.9	87.1
	28-31	855	8.4	35.2	56.4
	32–34	1,488	4.4	75	20.6
	35–36	1,067	1.6	85.7	12.8
Sex	Female	1,629	6	65.9	28.1
	Male	1,882	3.5	67.1	29.4
Birth weight (in grams)	<1,000	161	8.1	9.3	82.6
	1,000-<1,500	916	10.4	39.5	50.1
	1,500-<2,000	1,378	3.1	75.7	21.2
	>=2,000	1,056	1	86.8	12.1
Multiple birth	Yes	1,214	5	69	26
	No	2,297	4.4	65.3	30.3
C-Section	Yes	1,331	5.3	67.8	26.8
	No	2,180	4.2	65.8	30.1

The remaining 4.6% of the babies were still in the unit at the end of the study follow-up. Around 27% of babies were born at or below 32 weeks gestational age, of which 570 died, 307 were discharged alive and 79 were still in the unit. More than 69% of preterm babies weigh less than 2,000 grams. There were 1,077 babies born weighing less than 1,500 grams, of which 592 died, 377 were discharged alive and 108 were still in the unit.

Out of 1,882 male preterm infants 67.1% and 29.45% were discharged alive and died before discharge respectively. Among 1,629 female preterm infants 65.9% and 28.1% were discharged alive and died before discharge respectively. About 87.1% of preterm infants below 28 weeks of GA died before discharge from the hospital and 6.9% were still in NICU. About 12.8% of preterm infants between 35 and 36 weeks of GA died prior to discharge from the hospital and 85.7% were discharged alive. 69% of women with multiple births discharged alive while 26% of the women were died prior to discharge.67.8% of mothers who gave birth by the C-section were discharged alive while 26.8% were died prior to discharge from the hospital.

**Figure 2:**
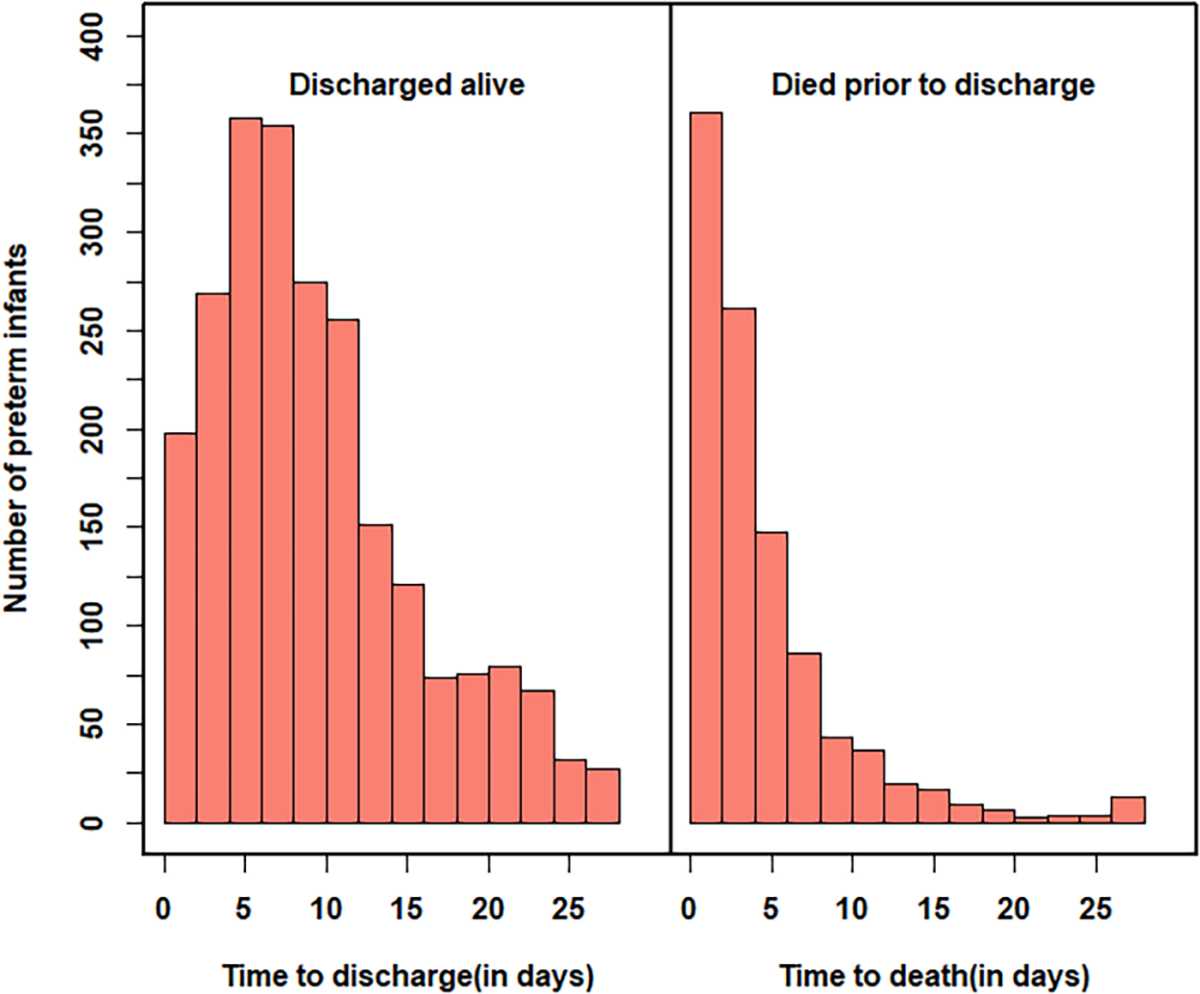
Distribution of time to discharge and time to death in the number of days.

The median LOS (death or discharge) was 7 days, with an interquartile range of eight days. Figure 2 depicts the distribution of times to discharged alive and times to death. The overall distribution of time to discharge was skewed to the right, with most infants discharged alive in the first two weeks and about 43% of the survivors were discharged in the first one week. Preterm babies who were dying in NICU had a median LOS of around 3 days, which indicates that half of the deaths occur in the first 3 days after birth. About 28.8% of the preterm infants died during their early neonatal periods (i.e., in the first week after birth). On the other hand, the median LOS was eighty days for those discharged alive.

**Figure 3:**
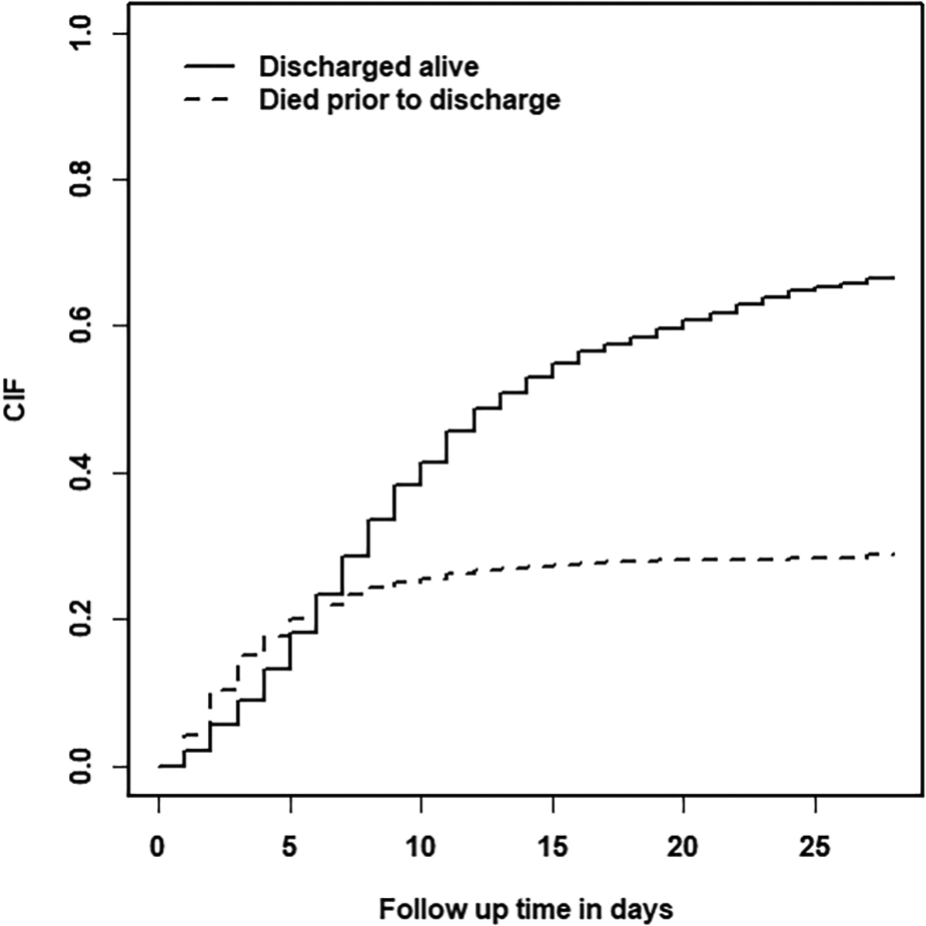
Plot of cumulative incidence of follow-up time.

The cumulative incidence function (CIF) was used to depict the likelihood of death in the hospital and discharge alive over time Figure 3 There was a steep rise in the probability of death for the first week of admission, which implies that the case fatality rate among preterm babies was higher during their early neonatal periods. On the other hand, the steepest rise in the probability of being discharged alive for preterm neonates was observed during the study follow-up. However, the odds of death remains lower than the odds of being discharged alive after one week of admission till the end of the study follow-up.

Table 2 presents a summary of common clinical diagnoses at the admission of preterm infants. Of the 3,511 preterm infants in the study, 1,595 (45.4%) were diagnosed with RDS and 1,312 (37.4%) were diagnosed with sepsis. The other common clinical conditions observed among preterm infants in this study were: apnea, pneumonia, anemia, hyperbilirubinemia, feeding problems, hypoglycemia, asphyxia, and NEC. About 45% of the infants diagnosed with RDS were discharged alive before the end of the study follow-up with median LOS of 10 days.

**Table 2:** Length of hospital stays of preterm infants by their common admission diagnosis.

Variables	# of Preterm infants	% Discharged Alive before the end of the follow-up	Length of stay		
			Mean	Median	IQR
RDS	1,595	45.45	11.23	10.00	9.00
Apnea	425	13.88	9.80	7.00	12.00
Sepsis	1,312	58.61	10.80	9.00	7.00
Pneumonia	95	48.42	11.93	11.00	6.50
Anemia	315	47.3	13.24	12.00	10.00
Hyperbilirubinemia	1,082	77.54	11.55	11.00	8.00
Feeding problem	809	57.35	10.89	9.00	9.00
Hypoglycemia	851	66.39	11.43	11.00	8.00
Asphyxia	248	29.84	10.34	10.00	8.75
Necrotizing enterocolitis	147	31.29	15.96	17.00	9.00

With a median duration of stay of 7 days, only 14% of the newborns diagnosed with apnea were discharged alive before the end of the study's follow-up. The majority of the preterm infants admitted with the clinical conditions hyperbilirubinemia and hypoglycemia were discharged alive with an approximate median duration of stay of 11.5 days.

For variable selection, the stepwise regression procedure in the forward or backward direction using the R package “crrstep” was used. All the aforementioned demographic and clinical characteristics listed in Tables 1, 2 were checked for their candidacy for the final statistical model. Based on these candidate predictors, the final Fine and Gray model using the R package “crr” was fitted. The Sub-Distributed-Hazard ratios with their 95% confidence intervals and *P*-values resulting from the final Fine and Gray model with all candidate predictors are shown in Table 3. All the demographic and clinical factors were found to be significantly associated with a longer LOS in the preterm infant population. Preterm newborns with low gestational age and birth weight had a greater likelihood of having a prolonged LOS. Complications from the clinical conditions of RDS, apnea, sepsis, pneumonia, anemia, asphyxia, and NEC in preterm infants significantly increase their LOS.

**Table 3:** Analysis of time to discharge, using a fine and gray competing-risks method to obtain a sub-distribution-hazard ratio.

Factors	Categories	Sub-distributed-hazard		
		SDH ratio	95% CI	*P*-Value
Gestational age (in weeks)	<28	Ref.	Ref.	Ref.
	28–31	3.449	(1.559, 7.630)	0.002
	32–34	5.520	(2.496, 12.207)	<0.001
	35–36	6.087	(2.741, 13.518)	<0.001
Birth weight (in grams)	<1,000	Ref.	Ref.	Ref.
	1,000-<1,500	2.827	(1.719, 4.648)	<0.001
	1,500-<2,000	5.713	(3.477, 9.386)	<0.001
	>=2,000	8.008	(4.843, 13.240)	<0.001
Respiratory distress syndrome		0.543	(0.495, 0.595)	<0.001
Apnea		0.241	(0.183, 0.318)	<0.001
Early onset neonatal sepsis		0.638	(0.587, 0.694)	<0.001
Pneumonia		0.564	(0.433, 0.736)	<0.001
Anemia		0.715	(0.614, 0.833)	<0.001
Asphyxia		0.351	(0.275, 0.450)	<0.001
Necrotizing enterocolitis		0.442	(0.338, 0.578)	<0.001

The cumulative incidence of discharge increased with an increase in gestational age and birth weight. In relation to preterm infants with a gestational age lower than 28 weeks, the sub-distribution hazard of discharge was 3.4 times higher for those infants with 28–31 weeks of gestation, 5.5 times higher for infants with 32–34 weeks of gestation, and 6.1 times higher for infants with 35–36 weeks of gestation. The discharge sub-distribution hazards for preterm neonate with birth weights (in grams) of 1,000 to less than 1,500, 1,500 to less than 2,000, and 2,000 and above were 2.8, 5.7, and 8.0 times higher, respectively, than for preterm neonate with birth weights of 1,000 and less. The rate of discharge decreased by 45.7% with the development of RDS (SDH ratio: 0.543), by 75.9% with the development of apnea (SDH ratio: 0.241), by 36.2% with sepsis (SDH ratio: 0.638), and by 43.6% with pneumonia (SDH ratio: 0.564). Similarly, the sub-distribution hazard of discharge decreased by 28.5% for infants diagnosed with anemia (SDH ratio: 0.715), by 64.9% for infants diagnosed with asphyxia (SDH ratio: 0.351) and by 55.8% for those infants diagnosed with necrotizing enterocolitis (SDH ratio: 0.442).

## Discussion

The capacity to accurately estimate LOS in the neonatal ICU has become more essential in recent years. As newborn survival rates have improved, so has the number of babies who require extended hospitalizations in the neonatal unit. However, there has been limited study on how to estimate LOS and what variables are necessary to take into account. The current study conducted a systematic review of the literature to determine what factors should be addressed in future LOS assessments.

Numerous studies have looked at predicting LOS in preterm newborns admitted to the NICU. In this study, the occurrence of Respiratory Distress Syndrome, Apnoea, Neonatal Sepsis, Pneumonia, Anaemia, Asphyxia, low birth weight, lower gestational age, and Necrotizing Enterocolitis were significant predictors of prolonged hospital LOS among preterm newborns in the NICU.

In our study, 66.6% of preterm babies survived and were discharged alive; the deathrate was 28.8%, in comparison 10.1% in the Saudi study and 12.8% in the Indian study. These disparities can be explained by the fact that newborn survival varies with healthcare quality.

Low birth weight and gestational age were the most important variables associated with longer LOS among neonates. The median LOS was 7 days for babies delivered between 23and36 weeks of gestation, which is same with a study conducted in Ethiopia on Children Admitted to the NICU (13). However, another study in Saudi Arabia reported a median LOS of 14.5 days for preterm infants admitted to the NICU. This inconsistency might be attributed from the variation in the gestational age and quality of care of admitted babies to the neonatal unit. The median LOS for the preterm infants with a gestational age of 28, 28–31, 32–34, and 35–36 weeks were 15.5, 12,9, and 7 days, respectively. On the other hand, the median LOS with a birth weight of less than 1 kg, 1–1.5 kg, 1.5–2 kg, and 2 kg and above were 14, 13, 9, and 7 days, respectively. This result showed that the LOS decreased as the weeks of gestational age and birth weight increased. These findings are supported by the study on very early preterm infants (25 weeks to 33 weeks of gestation) (14). A similar study which includes preterm infants from 24 to 31 gestational age also concludes that babies born at an early gestational age (24 and 25 weeks) who are discharged alive have a median LOS slightly longer than the interval to their estimated date to delivery and the babies discharged sooner when they are born at 30 and 31 weeks (6).

Birth weight influences the duration of hospital stay with an SDH ratio of 2.8, 5.7, and 8 times higher for preterm infants weighing 1kg−1.5 kg, 1.5–2 kg, and ≥2 kg as compared to preterm newborn weighing below1kg grams, respectively. The inverse association of our finding between gestational age and LOS was similar to the study in Nigeria which concluded the LOS increased with decreasing birth weight and gestational age (15). Several studies also revealed a negative correlation between birth weight and gestational age with LOS (14–17).

Preterm babies with complicated clinical conditions will prolong their length of stay in the hospital. In the present study, preterm infants with respiratory distress syndrome (RDS) and sepsis had greater incidence of LOS which is not unexpected finding in our analysis as it is already known that RDS and sepsis increase LOS among hospitalized preterm infants (14). A similar study done in Ethiopia on the factor affect LOS in neonatal sepsis found that presence comorbidities, neurological problems low birth weight are associated with prolonged length of stay in neonatal ICU (18).Moreover, necrotizing enterocolitis (NEC) can potentially prolong their LOS, which is consistent with the study in the US (19). An anaemic preterm infant has been associated with a reduced likelihood of discharge. Similarly, preterm infants with early onset sepsis were also associated with a decrease in the incidence of discharge with sub-distribution hazard of 0.715 and 0.638, respectively. A study from Nigeria also showed that anaemia and sepsis were also found to be substantially related with a longer length of stay (20). Again, our result is also supported by the study in Eritrea (16) concerning the significance of pneumonia in prolonging the hospital length of stay.

### Limitation of study

The study has limitations in analyzing the clinical conditions which are a different clinical staging, disease severity, and complication, such as NEC, RDS, and anemia which have an impact on LOS. The readmission information and maternal antenatal steroids treatment not assessed due to limited information sources.

## Conclusions

For planning to improve service efficiency, the cumulative incidence of being discharged alive should be assessed by incorporating time to death. LOS in the NICU for preterm infants is influenced by both demographic and clinical characteristics. The cumulative incidence of discharge was increased for every increase in gestational age and birth weight. Having complications from the clinical conditions of RDS, apnea, sepsis, pneumonia, anaemia, asphyxia, and NEC in preterm infants significantly increases their hospital LOS.

## Data Availability

The original contributions presented in the study are included in the article/Supplementary Material, further inquiries can be directed to the corresponding author.
